# FLASH reduces radiation-induced oral mucositis in a mouse model of Fanconi anemia

**DOI:** 10.1016/j.ctro.2026.101334

**Published:** 2026-10-23

**Authors:** Phoebe Loo, Margaret Pan, Man Zhao, Stavros Melemenidis, Joseph B. Schulz, Dixin Chen, Lucy Whitmore, Sara Richter, Frederick M. Dirbas, Kerriann M. Casey, Edward E. Graves, Michael W. Epperly, Joel Greenberger, Billy W. Loo, Erinn B. Rankin

**Affiliations:** a Department of Radiation Oncology, Stanford University School of Medicine, Stanford, CA, 94305, United States of America; b Department of Surgery, Stanford University School of Medicine, Stanford, CA, 94305, United States of America; c Department of Obstetrics and Gynecology, Stanford University School of Medicine, Stanford, CA, 94305, United States of America; d Department of Comparative Medicine, Stanford University School of Medicine, Stanford, CA, 94305, United States of America; e Stanford Cancer Institute, Stanford University School of Medicine, Stanford, CA, 94305, United States of America; f Department of Radiation Oncology, University of Pittsburgh, Pittsburgh, PA, 15260, United States of America

**Keywords:** radiation therapy, FLASH, oral mucositis, DNA damage

## Abstract

This study demonstrates that FLASH radiotherapy reduces oral mucositis in both wild-type and *Fanca*-deficient mice. At the cellular level, FLASH attenuated radiation-induced inflammation in the tongue relative to conventional irradiation in both genotypes, indicating that FLASH mitigates inflammatory processes associated with oral mucositis. Collectively, these findings support the potential of FLASH radiotherapy to reduce radiation-induced oral mucositis in the context of *Fanca* deficiency and the Fanconi anemia genomic background.

## 1 Introduction

Ultra-rapid FLASH radiotherapy (FLASH) is emerging as an exciting new treatment paradigm to enhance the therapeutic index of radiotherapy. In preclinical studies, FLASH reduces radiation-induced toxicities in multiple tissues including the lung [1], intestine [2], [3], skin [4], [5], brain [6], [7] and oral cavity [8] compared to conventional radiotherapy (CONV), in the 2–3 orders of magnitude lower delivery rates used clinically, while demonstrating comparable tumor control [1], [9]. These findings have generated considerable interest in the potential clinical translation of FLASH as a strategy to expand the therapeutic index of radiotherapy with clinical trials ongoing and in development [10]. However, it remains unknown whether these benefits extend to patient populations with intrinsic radiosensitivity.

One such radiosensitive population is patients with Fanconi anemia (FA), an autosomal recessive disorder caused by germline mutations in any of at least 22 FA genes [11], [12], which collectively play critical roles in DNA repair [13], [14], [15]. FA is characterized by cellular hypersensitivity to DNA crosslinking agents [16] and a markedly increased risk of cancer, with a 50-fold higher incidence of solid tumors and a 500-fold higher incidence of head and neck squamous cell carcinomas (HNSCC) compared to the general population [17], [18], [19]. As survival of FA patients has improved through hematopoietic stem cell transplantation, the incidence of malignancies such as HNSCC has become an increasing clinical concern [17]. Since FA patients are highly sensitive to DNA damaging agents, radiotherapy (a key component of standard HNSCC treatment) is often limited by severe normal tissue toxicities including mucositis, dysphagia, and pancytopenia, which can compromise treatment efficacy and overall survival [20], [21], [22]. These findings highlight the unmet clinical need for improved treatment strategies to enhance the therapeutic index of radiotherapy for the treatment of solid tumors that develop in FA patients.

The most common FA mutation occurs in the *FANCA* gene, which encodes a protein that functions in the nucleus to coordinate multiple repair processes such as DNA lesion recognition, incision, bypass, and repair, and interacts with other pathways including homologous recombination [12], [23], [24], [25], [26]. Given its clinical prevalence and milder phenotype relative to other FA mutations [13], *Fanca*-deficient (*Fanca*^−/−^) mouse models provide a clinically relevant model for studying radiosensitivity and strategies to mitigate normal tissue injury during radiotherapy. Here we investigate FLASH as a strategy to reduce normal tissue toxicities associated with oral cavity irradiation in *Fanca*^−/−^ mice.

## 2 Materials and methods

### 2.1 Study design

The objective of this study was to investigate the normal tissue-sparing effect of FLASH compared to conventional dose rate (CONV) irradiation to the oral cavity in *Fanca*^*−/−*^ mice. All procedures for animal care and use were approved by the Institutional Animal Care and Use Committee of Stanford University in accordance with institutional and NIH guidelines. Cohorts of *Fanca*^+/+^ and *Fanca*^−/−^ mice (129/Sv) [27] were generated from *Fanca*^+/+^ and *Fanca*^−/−^ breeding pairs [20] and irradiated with both FLASH and CONV modalities. Male mice were selected given the higher incidence of HNSCC in males [28], while a single-sex cohort was used to minimize potential variability associated with sex-dependent physiological differences, including those documented in murine submandibular salivary glands [29]. Male mice aged 10–12 weeks were anesthetized with a mixture of ketamine (100 mg/kg) and xylazine (10 mg/kg) by intraperitoneal injection. Control mice underwent sham irradiation under the same experimental conditions as irradiated mice, except for radiation exposure. The toxicity of FLASH compared to CONV was evaluated in non-tumor bearing mice. *Fanca*^+/+^ and *Fanca*^−/−^ mice were irradiated with 26 and 18 Gy, respectively, of FLASH or CONV, and tongues were harvested at 12 h (hpi) and 10 days (dpi) post-irradiation. Radiation dose and tissue-harvesting timepoint were selected based on pilot study data, with genotype-specific doses used to optimize visualization of radiation-induced ulceration while minimizing mortality in *Fanca*^+/+^ and the more radiosensitive *Fanca*^−/−^ backgrounds (Supp. Fig. 1). These genotype-specific doses were selected to evaluate the effects of FLASH relative to CONV within each genotype, rather than directly compare the magnitude of FLASH-mediated protection between genotypes. Tongues were stained with toluidine blue and sectioned for histological analysis of oral mucositis. Immunohistochemistry was also performed on tongue sections to evaluate toxicity on a molecular level.

**Fig. 1:**
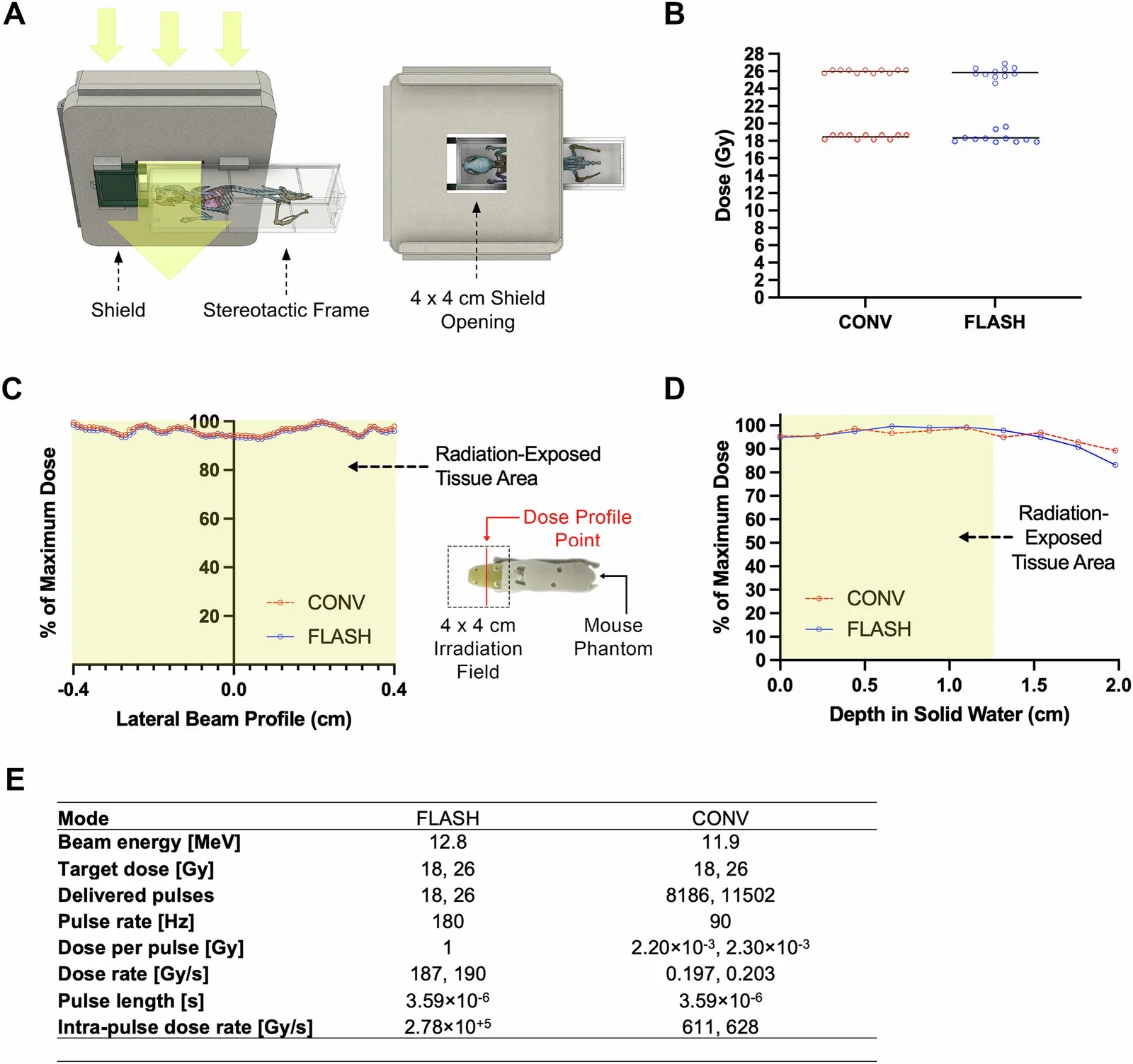
Experimental configuration for ultra-high dose rate FLASH and conventional oral cavity irradiation. (A) 3-D design of irradiation shield and stereotactic frame for reproducible mouse positioning within the irradiation field. Shield consists of 3-D printed PLA plastic shell filled with 1.5 cm thick Cerrobend to limit dose delivery from a nominal 12 MeV electron beam to within shield opening. 4 cm × 4 cm shield opening encompasses the entire oral cavity region, extending from the nose to the first rib. (B) Measured doses delivered, which match target values of 18 and 26 Gy for both FLASH and CONV. (C—D) Lateral and dorsoventral beam profiles recorded using radiochromic films. Highlighted regions indicate the projected tissue area within the irradiation field. Doses are uniformly distributed within the oral cavity region. (C) Dose readings within the irradiation field (dashed box) were acquired at indicated positions (red line) on radiochromic film placed in a coronally-sectioned 3-D printed mouse phantom during irradiation. (E) Geometric and beam parameters for FLASH and CONV. Higher pulse rate and dose per pulse in FLASH results in an average dose rate ~ 940 times that of CONV. (For interpretation of the references to colour in this figure legend, the reader is referred to the web version of this article.)

Further details regarding experimental procedures and analyses are provided in the Supplemental Materials and Methods [20], [27], [30], [31], [32], [33], [34], [35].

## 3 Results

### 3.1 FLASH irradiation decreases radiation-induced oral mucositis in *Fanca*^+/+^ and *Fanca*^−/−^ mice

To compare the toxicity of FLASH and conventional oral cavity irradiation in the *Fanca*^+/+^ and *Fanca*^−/−^ backgrounds, we used a clinical linear accelerator to deliver reproducible and homogenous lateral and depth dose throughout the mouse oral cavity in FLASH (190 Gy/s) and CONV (0.2 Gy/s) modalities (Fig. 1A-E).

To evaluate the radiosensitivity of *Fanca*^−/−^ mice compared to *Fanca*^+/+^ controls, we first performed a pilot study using oral cavity irradiation. Our analysis focused on radiation-induced oral mucositis (RIOM), as it represents a primary dose-limiting toxicity in HNSCC patients characterized by acute pain, dysphagia, opioid dependence, and weight loss [36]. Preliminary toxicity assays demonstrated that *Fanca*^−/−^ mice exhibit increased sensitivity to oral irradiation; specifically, 75% (6/8) of the *Fanca*^−/−^ mice met early euthanasia criteria within 10 days post 26 Gy irradiation, whereas 0% (0/8) of the *Fanca*^+/+^ group reached those endpoints (Supp. Fig. 1). Furthermore, we found that 18 Gy in *Fanca*^−/−^ mice induced a severity of oral mucositis comparable to 26 Gy in *Fanca*^+/+^ mice, without resulting in acute morbidity (Supp. Fig. 1). Therefore, we utilized 26 Gy for the *Fanca*^+/+^ background and 18 Gy for the *Fanca*^−/−^ background to evaluate the normal tissue effects of FLASH relative to conventional oral cavity irradiation within each genotype.

To investigate the safety of FLASH irradiation to the head and neck region, we delivered 26 or 18 Gy FLASH and CONV to the oral cavity of *Fanca*^+/+^ and *Fanca*^−/−^ mice and analyzed the tongues for toxicity at 10 days post-irradiation (dpi). In the *Fanca*^+/+^ background, FLASH and CONV-irradiated mice demonstrated a similar decline in body weight following irradiation (Supp. Fig. 2A). Notably, in the *Fanca*^−/−^ background, 4/7 FLASH-irradiated mice started to gain weight by 10 dpi, while all CONV-irradiated mice continued to lose weight (Supp. Fig. 2B). Oral ulceration severity, quantified as the fraction of toluidine-blue stained tongue area relative to total tongue area, was reduced with FLASH compared to CONV in both *Fanca*^+/+^ and *Fanca*^−/−^ mice (Fig. 2A-C). Histological analyses of the tongues using an established scoring system revealed *Fanca*^+/+^ CONV-irradiated tongues scored higher for mucositis severity with 100% of mice with a grade 3 or 4 lesion compared to 43% of mice that received FLASH irradiation (Fig. 2D-F, Sup. Fig. 3). In the *Fanca*^−/−^ mice, 100% of CONV-irradiated tongues developed a grade 3 or 4 lesion compared to 57% of FLASH-irradiated tongues that developed a grade 3 or 4 lesion. To further characterize the histopathologic response, we evaluated inflammatory cell infiltration based on the extent and depth of inflammation within tongue sections. FLASH-irradiated tongues exhibited lower cumulative inflammation scores compared to CONV-irradiated tongues in both *Fanca*^+/+^ and *Fanca*^−/−^ mice (Fig. 2G-H). Together these data indicate that FLASH irradiation reduces radiation-induced tongue injury in both *Fanca*^+/+^ and *Fanca*^−/−^ mice compared to CONV irradiation.

**Fig. 2:**
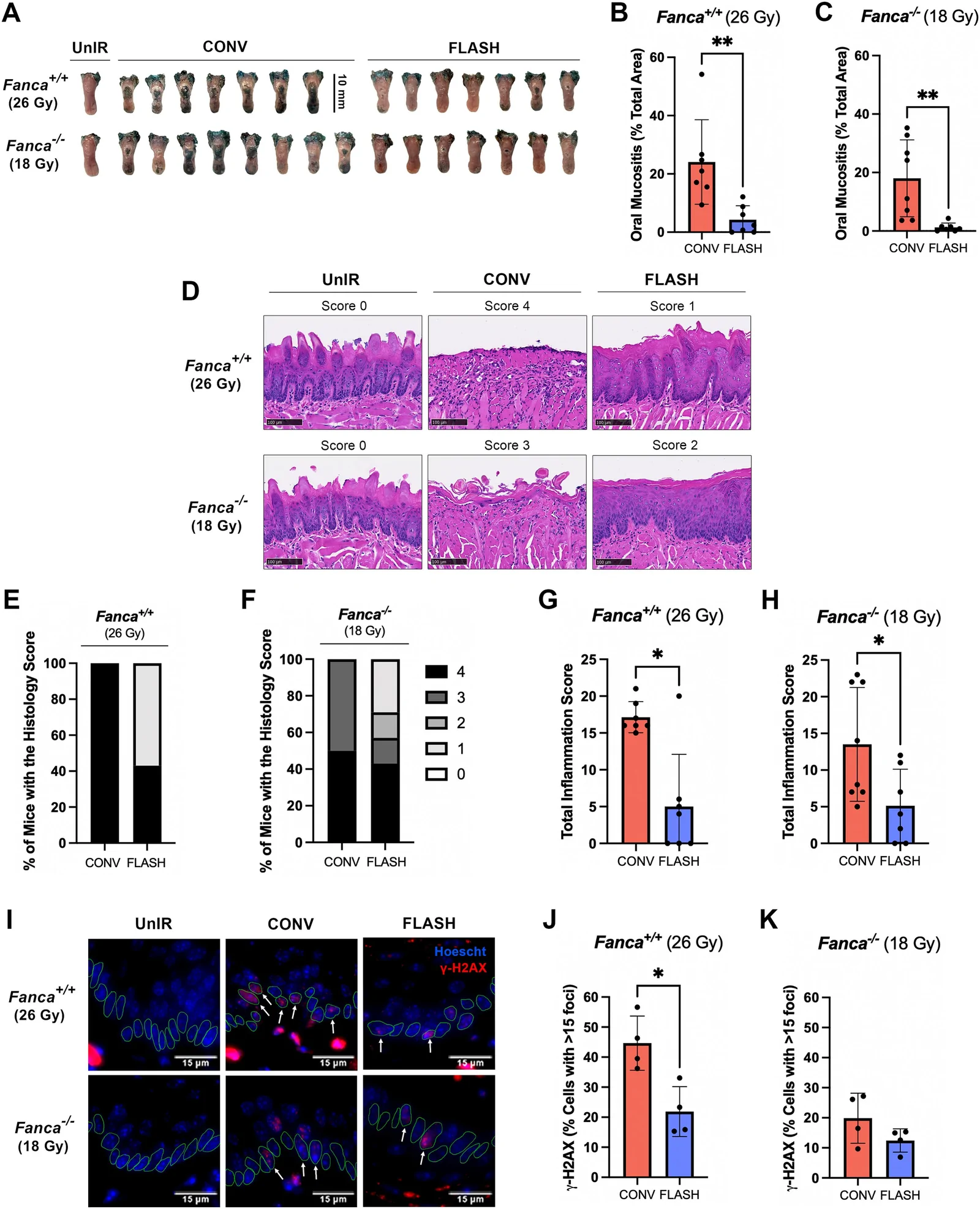
FLASH reduces oral mucositis compared to CONV in Fanca^+/+^ and Fanca^−/−^ mice. Fanca^+/+^ and Fanca^−/−^ mice were irradiated with 26 and 18 Gy, respectively, of CONV or FLASH and tongues were harvested at 10 dpi. For Fanca^+/+^, UnIR n = 5, CONV n = 7, and FLASH n = 7. For Fanca^−/−^, UnIR n = 5, CONV n = 8, and FLASH n = 7. (A) Tongues were stained with toluidine blue to visualize ulceration. Not all UnIR tongues are pictured. UnIR = Unirradiated. dpi = days post-irradiation. (B—C) Ulcerated area expressed as a percentage of total tongue area, quantified in digital photographs using Fiji ImageJ. Data presented separately for each genotype. Each data point represents the average of measurements from three independent scorers (** p < 0.01). Scale bar represents 10 mm. UnIR tongues exhibited no ulceration. (D) Representative tongue epithelium images from H&E stained tongue sections used for histopathologic evaluation. Higher scores indicate more severe toxicity. (E-F) Percentage of mice in each group with a maximum histopathologic score of 0, 1, 2, 3, or 4 (** p < 0.01). Data presented separately for each genotype. (G-H) Quantification of inflammatory infiltration in H&E-stained tongue sections, scored based its extent and depth. Data presented separately for each genotype. Each data point represents the cumulative inflammation score for one mouse, calculated as the sum of scores from six tongue sections (* p < 0.05). (I) Representative γ-H2AX (red) and Hoesch (blue) stained images of tongue epithelia from irradiated and unirradiated Fanca^+/+^ and Fanca^−/−^ mice at 12 h post-irradiation (hpi). Arrows indicate basal epithelial cells (outlined in green) with >15 γ-H2AX foci. (J-K) Quantification of positive γ-H2AX staining, represented by the percentage of total basal epithelial cells with >15 punctate γ-H2AX foci. Data presented separately for each genotype. n = 4 mice per group. Unirradiated tongues exhibited no positive γ-H2AX basal epithelial cell staining. Three images were analyzed per mouse, with an average of 70 epithelial cells analyzed per image. Values from the three images were averaged to generate a single value for each mouse (* p < 0.05). (For interpretation of the references to colour in this figure legend, the reader is referred to the web version of this article.)

Next, we investigated whether FLASH reduces basal cell DNA damage relative to CONV as a potential mechanism underlying its reported normal tissue-sparing effects. The proliferative basal cell layer of the tongue epithelium is essential for epithelial maintenance and regeneration following injury and is particularly radiosensitive due to its high mitotic activity [37]. Consequently, damage in this compartment disrupts tissue repair, contributing to acute and chronic oral toxicities. At 12 h post-irradiation (hpi), regardless of genetic background, unresolved DNA damage was evident in tongue basal epithelial cells of both CONV and FLASH-irradiated mice, as indicated by γ-H2AX staining, whereas unirradiated controls showed no detectable signal (Fig. 2I). Within *Fanca*^+/+^ mice, the percentage of basal epithelial cells containing greater than 15 punctate γ-H2AX foci was increased in CONV-irradiated tongues compared to FLASH-irradiated counterparts (Fig. 2J-K). *Fanca*^*−/−*^ mice demonstrated a similar trend, with CONV-irradiated tongues exhibiting a higher percentage of γ-H2AX positive basal epithelial cells compared to FLASH-irradiated counterparts, although the difference did not reach statistical significance. These data suggest that FLASH irradiation reduces DNA damage compared to CONV irradiation in the basal cell layer of the tongue epithelium of wild type mice.

## 4 Discussion

Here we demonstrate that FLASH mitigates radiation-induced oral mucosal toxicity in both *Fanca*^+/+^ and *Fanca*^−/−^ mice, indicating that the normal tissue-sparing properties of FLASH may extend to the setting of intrinsic radiosensitivity.

Oral mucositis in the tongue epithelium is driven largely by injury to the proliferative basal epithelial compartment, where radiation-induced cellular injury, including DNA damage, disrupts epithelial renewal and impairs barrier integrity. In line with this pathology, we observed reduced ulceration, improved histopathologic architecture, lower mucositis severity scores, and reduced inflammatory infiltration in *Fanca*^+/+^ mice and *Fanca*^−/−^ mice following irradiation with FLASH compared to CONV. Consistent with our findings in *Fanca*^+/+^ mice using a high-energy electron beam, a recent preclinical study using a proton beam demonstrated reduced tongue ulceration and improved histopathologic architecture with FLASH compared to standard dose rate proton therapy [8], suggesting that this effect is preserved across radiation types. The phenotypic differences observed in the present study were accompanied by a reduction in γ-H2AX burden within basal epithelial cells of wild type mice at 12 h post-irradiation (hpi) with FLASH. As γ-H2AX serves as a surrogate marker of DNA double-strand breaks, the reduced γ-H2AX signal following FLASH may reflect differences in initial damage induction and/or enhanced DNA repair processes that contribute to the observed tissue sparing in wild type mice, although further investigation is needed to distinguish between these possibilities. These findings align with prior reports demonstrating reduced unresolved γ-H2AX in normal tissues following isodoses of FLASH relative to CONV [2], [38], extending this observation to the basal epithelial compartment of the tongue. In contrast, reports examining earlier timepoints have yielded mixed results, with some studies observing reduced γ-H2AX signaling following FLASH while others report no significant differences relative to CONV [38], [39].

Notably, FLASH reduced oral mucositis in the *Fanca*^−/−^ background. *FANCA* plays a central role in the Fanconi anemia DNA repair pathway, and its loss confers heightened radiosensitivity due to impaired repair of DNA lesions [12]. FA proteins collaborate in the nucleus to coordinate multiple repair processes including DNA lesion recognition, incision, bypass, and repair [12], [24], and have been shown to interact with other DNA repair pathways such as nucleotide excision repair, translesion synthesis, and alternative end joining [25], [26]. Our findings demonstrate that FLASH irradiation maintains its ability to reduce the severity of radiation-induced oral mucositis in this background, supporting its potential to mitigate normal tissue toxicity even in intrinsically radiosensitive settings. Although genotype-specific radiation doses were selected to achieve measurable oral mucositis while minimizing morbidity, this experimental design does not permit direct comparisons of the magnitude of FLASH-mediated protection between *Fanca*^*+/+*^ and *Fanca*^*−/−*^ mice. The lack of statistical significance in FLASH-mediated γ-H2AX reduction within the *Fanca*^−/−^ cohort may stem from constraints in dose and timing rather than an absence of biological effect. While doses were empirically selected based on pilot data to induce comparable levels of tongue ulceration across genotypes, these parameters may not be optimal for resolving differences between FLASH and CONV for secondary endpoints. Future studies incorporating alternative radiation doses and temporal analysis of DNA repair kinetics would help determine whether differences between FLASH and CONV become more apparent in the *Fanca*^−/−^ background and provide insight into differences in initial DNA damage induction and subsequent damage resolution. Additional mechanistic investigations, including analyses of FANCD2 activation and nuclear foci formation, potentially alongside downstream repair markers such as RAD51, would help clarify the contribution of FA-mediated DNA repair to FLASH-mediated tissue sparing.

Collectively, these findings provide an foundation for investigation the potential applications of FLASH in intrinsically radiosensitive contexts. Patients with inherited DNA repair deficiencies, including those with Fanconi anemia, experience profound toxicity following conventional radiotherapy and often face limited therapeutic options. Strategies that expand the normal therapeutic window without compromising tumor control are therefore of particular importance in this population. Future studies incorporating tumor-bearing models will be critical to determine whether the normal tissue-sparing effects observed here are maintained without loss of anti-tumor efficacy. As only male mice were included in this study, further investigation is needed to determine whether these findings extend to female mice. While the present study demonstrates the protective effects of FLASH against early radiation-induced oral mucosal injury in a mouse model of Fanconi anemia, it remains to be determined whether these effects extend to chronic radiation-induced toxicity. Taken together, our data support continued preclinical evaluation of FLASH as a strategy to mitigate radiation-induced normal tissue injury in radiosensitive settings.

## 5 Conclusion

FLASH irradiation reduces acute radiation-induced oral mucosal toxicity in *Fanca*^+/+^ and *Fanca*^−/−^ mice. This tissue-sparing effect is accompanied by reduced inflammatory infiltration. Together, these findings provide a foundation for further investigation of FLASH-mediated normal tissue protection in intrinsically radiosensitive settings.

## CRediT authorship contribution statement

**Phoebe Loo:** Writing – review & editing, Writing – original draft, Visualization, Validation, Methodology, Investigation, Formal analysis, Data curation, Conceptualization. **Margaret Pan:** Writing – review & editing, Investigation. **Man Zhao:** Writing – review & editing, Writing – original draft, Investigation. **Stavros Melemenidis:** Writing – review & editing, Methodology, Investigation, Formal analysis. **Joseph B. Schulz:** Investigation. **Dixin Chen:** Writing – review & editing, Methodology, Data curation. **Lucy Whitmore:** Writing – review & editing, Methodology, Data curation. **Sara Richter:** Writing – review & editing, Formal analysis, Data curation. **Frederick M. Dirbas:** Writing – review & editing, Supervision. **Kerriann M. Casey:** Writing – review & editing, Supervision, Methodology, Formal analysis, Data curation. **Edward E. Graves:** Writing – review & editing, Supervision, Formal analysis, Data curation. **Michael W. Epperly:** Writing – review & editing, Resources, Methodology, Conceptualization. **Joel Greenberger:** Writing – review & editing, Resources, Investigation, Conceptualization. **Billy W. Loo:** Writing – review & editing, Supervision, Resources, Project administration, Funding acquisition, Formal analysis, Conceptualization. **Erinn B. Rankin:** Writing – review & editing, Writing – original draft, Validation, Supervision, Resources, Project administration, Funding acquisition, Formal analysis, Conceptualization.

## Declaration of competing interest

The authors declare the following financial interests/personal relationships which may be considered as potential competing interests: Billy W. Loo Jr. reports financial support was provided by National Institutes of Health. Billy W. Loo Jr. reports financial support was provided by Varian Medical Systems Inc. Billy W. Loo Jr. reports a relationship with TibaRay that includes: board membership. Billy W. Loo Jr. reports a relationship with BeiGene that includes: consulting or advisory. If there are other authors, they declare that they have no known competing financial interests or personal relationships that could have appeared to influence the work reported in this paper.
